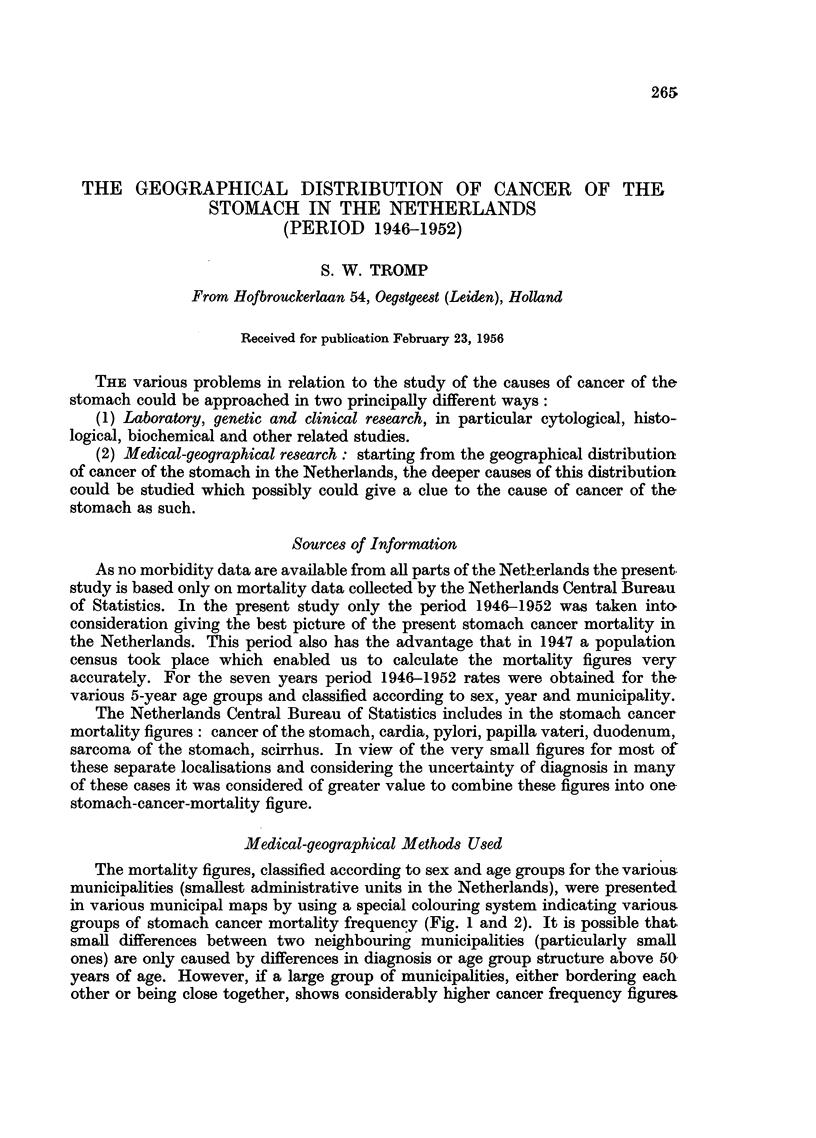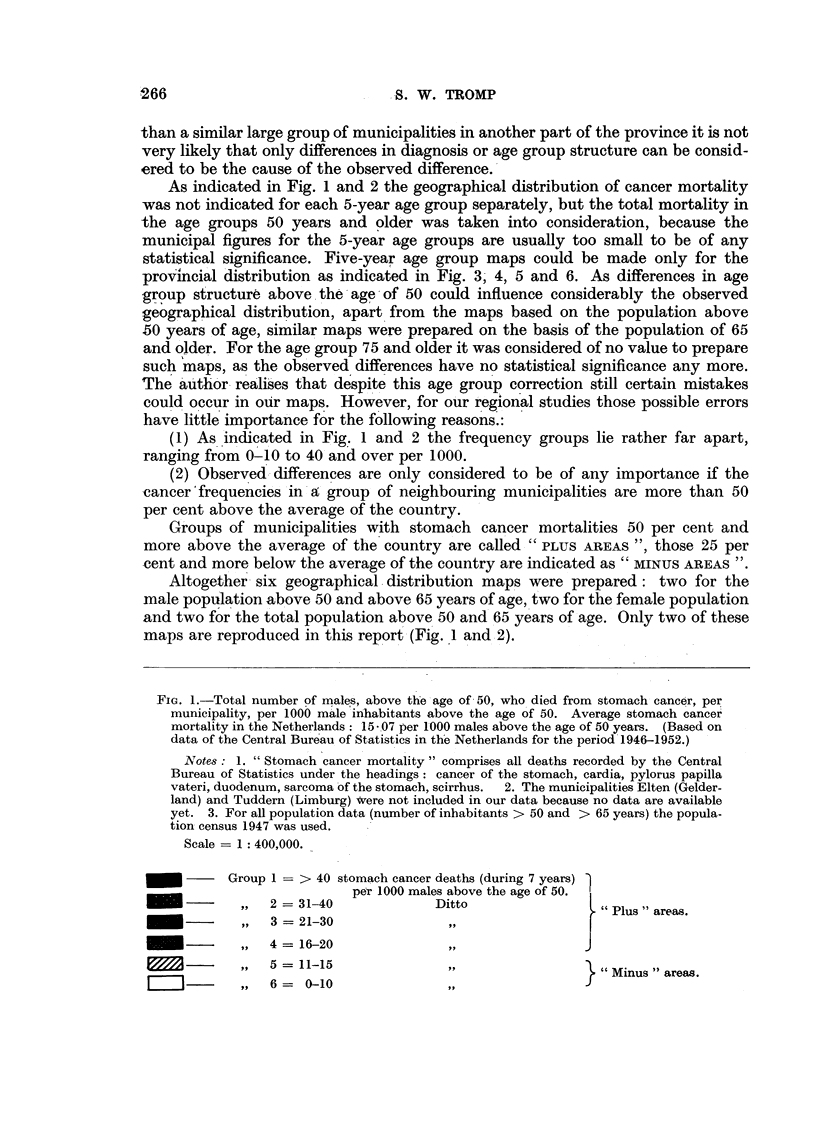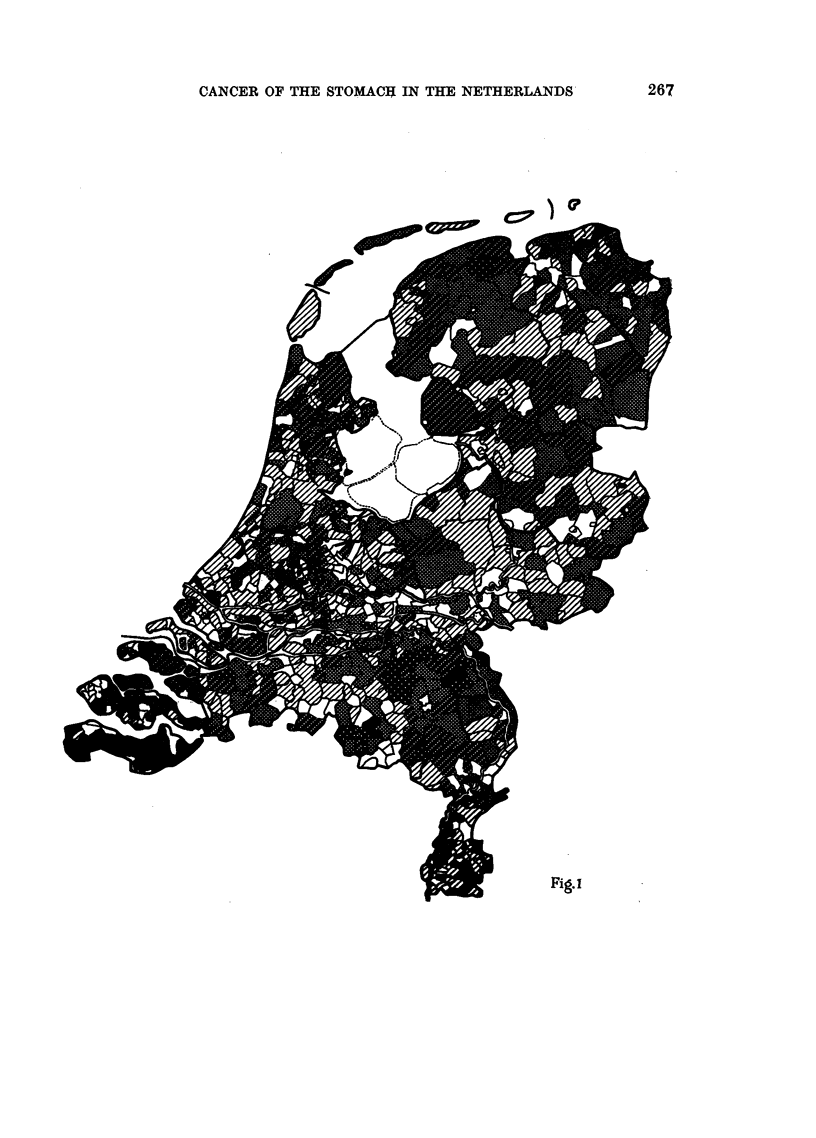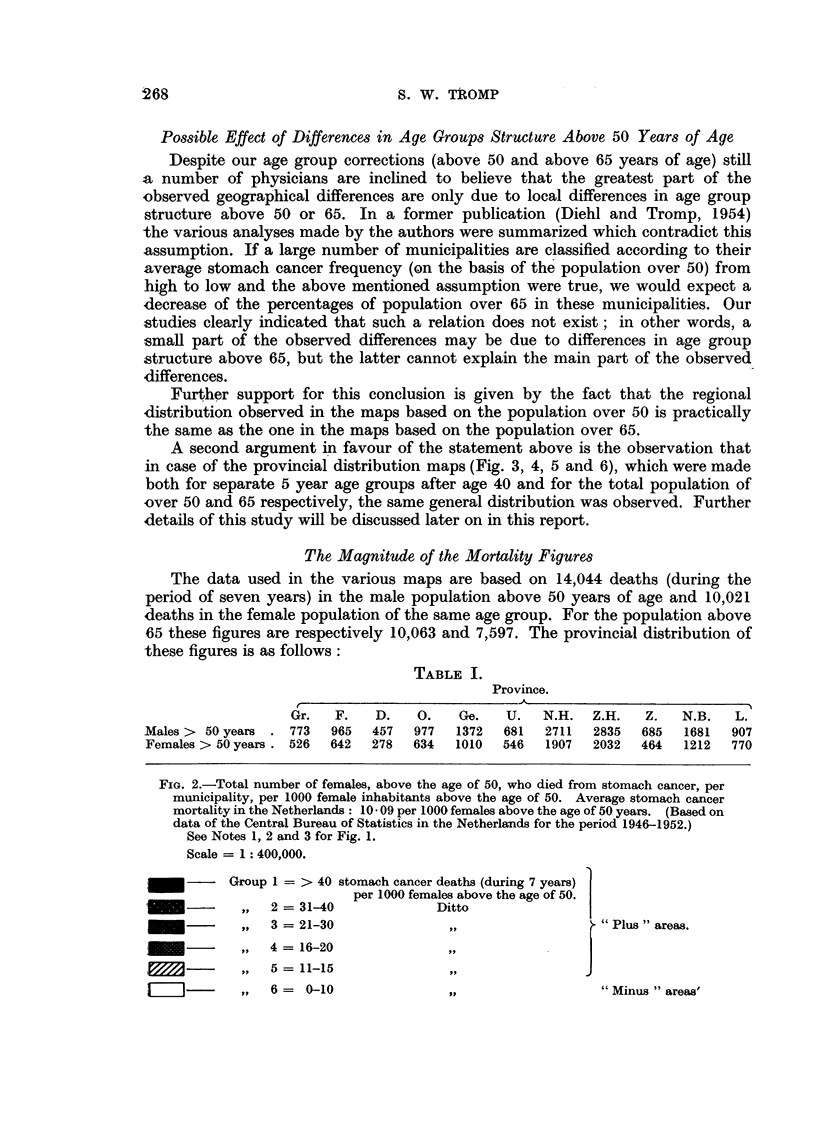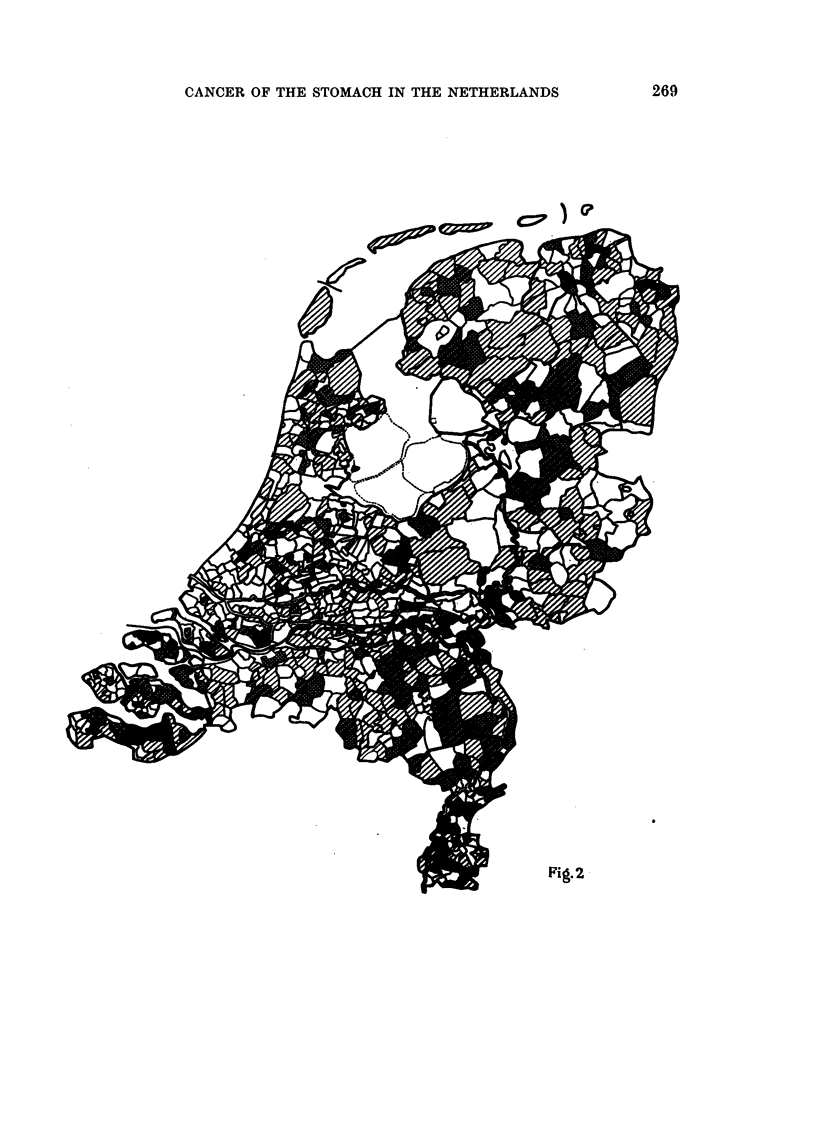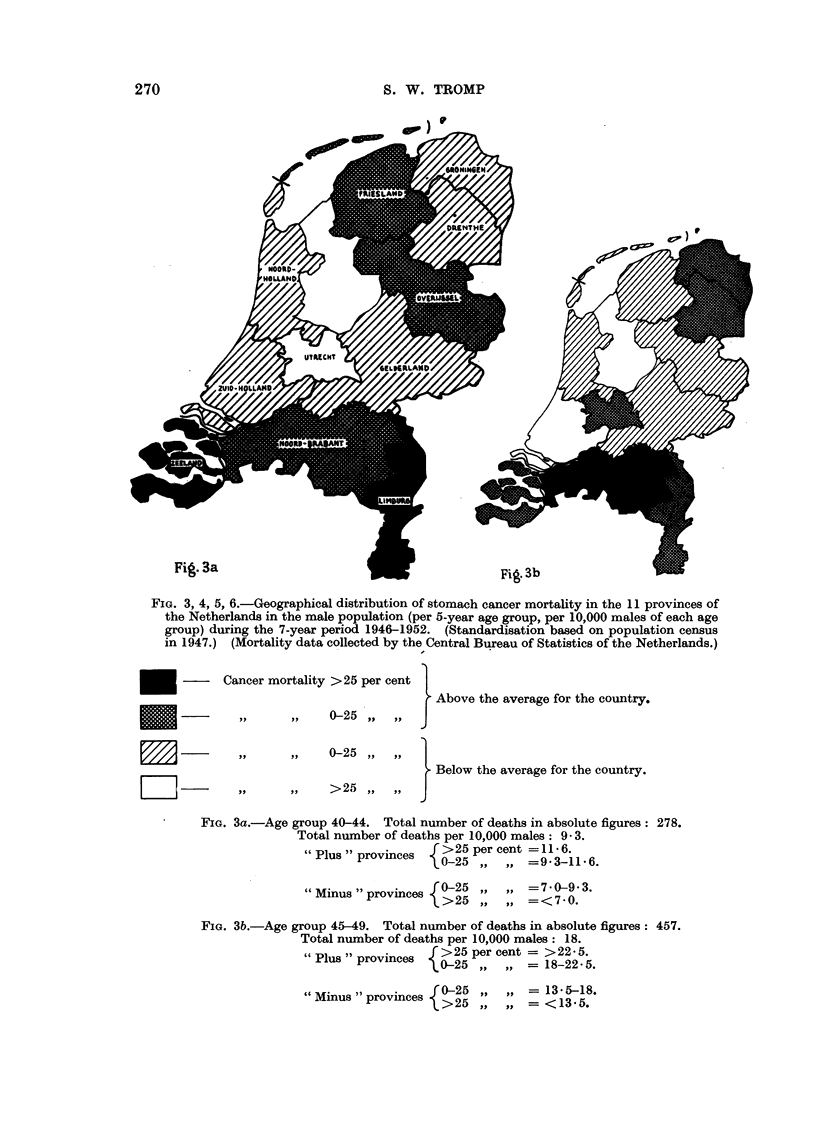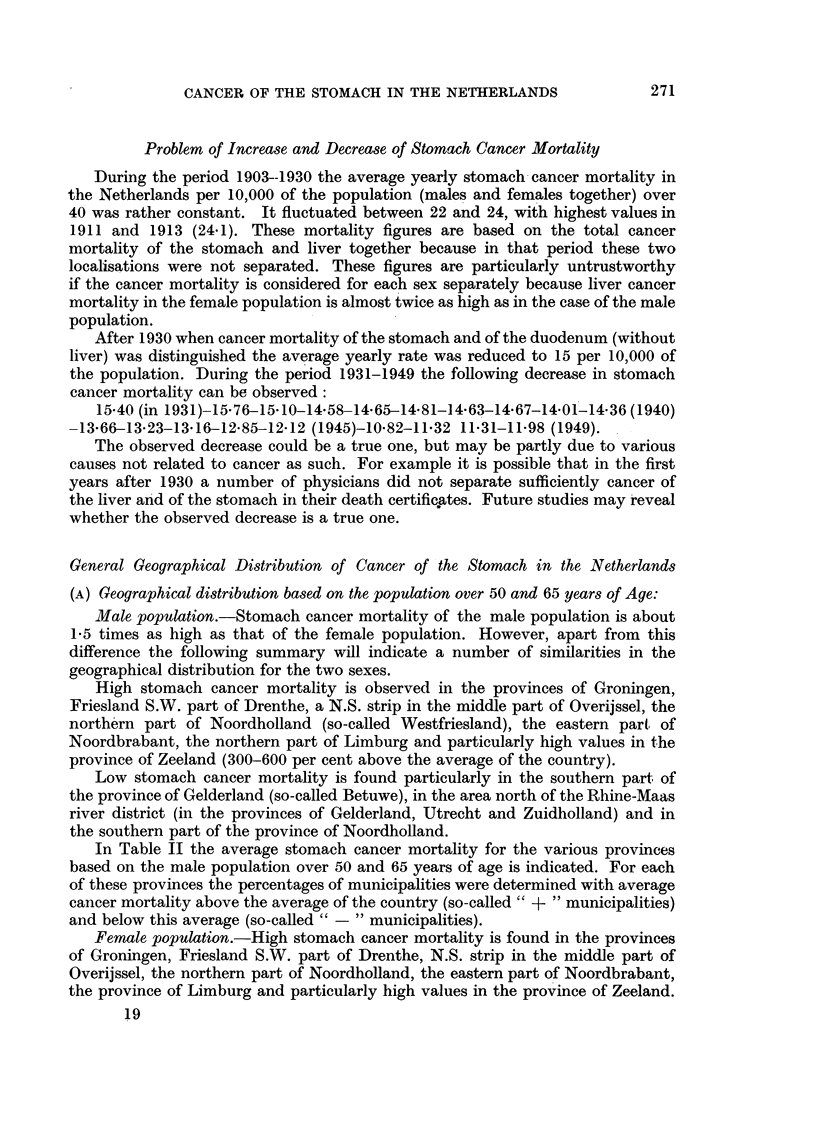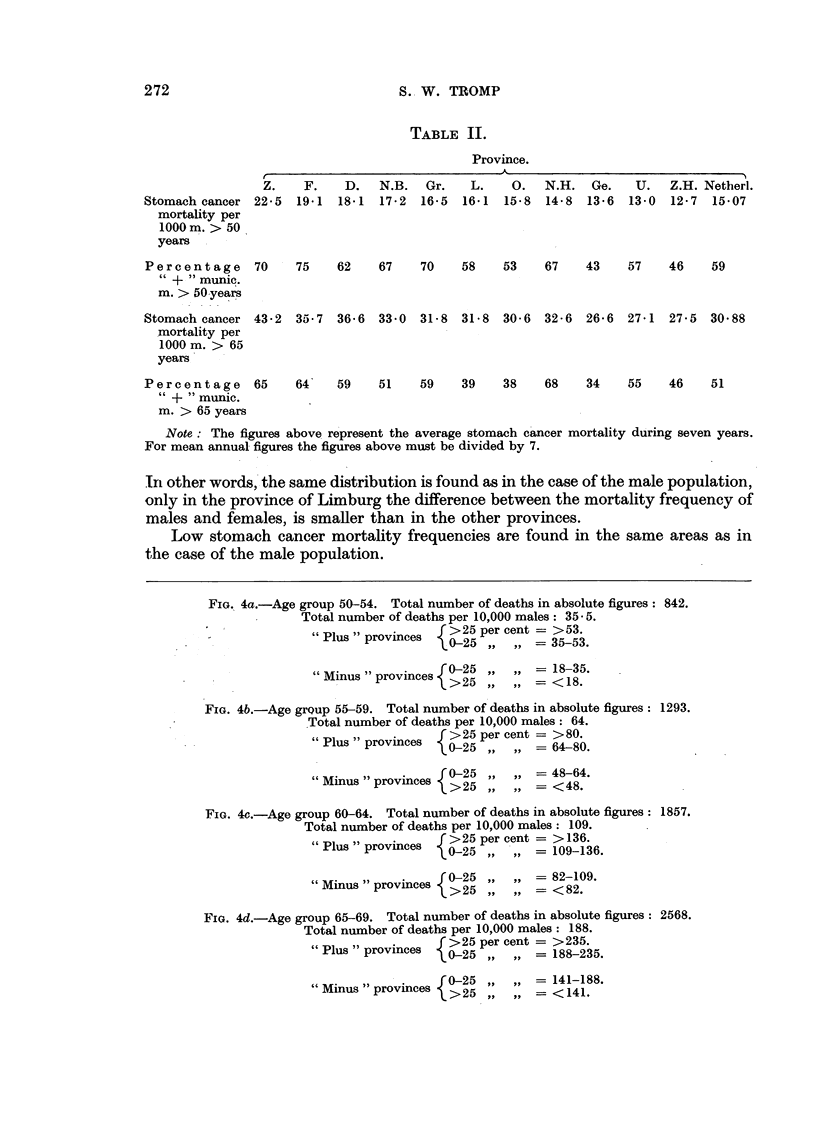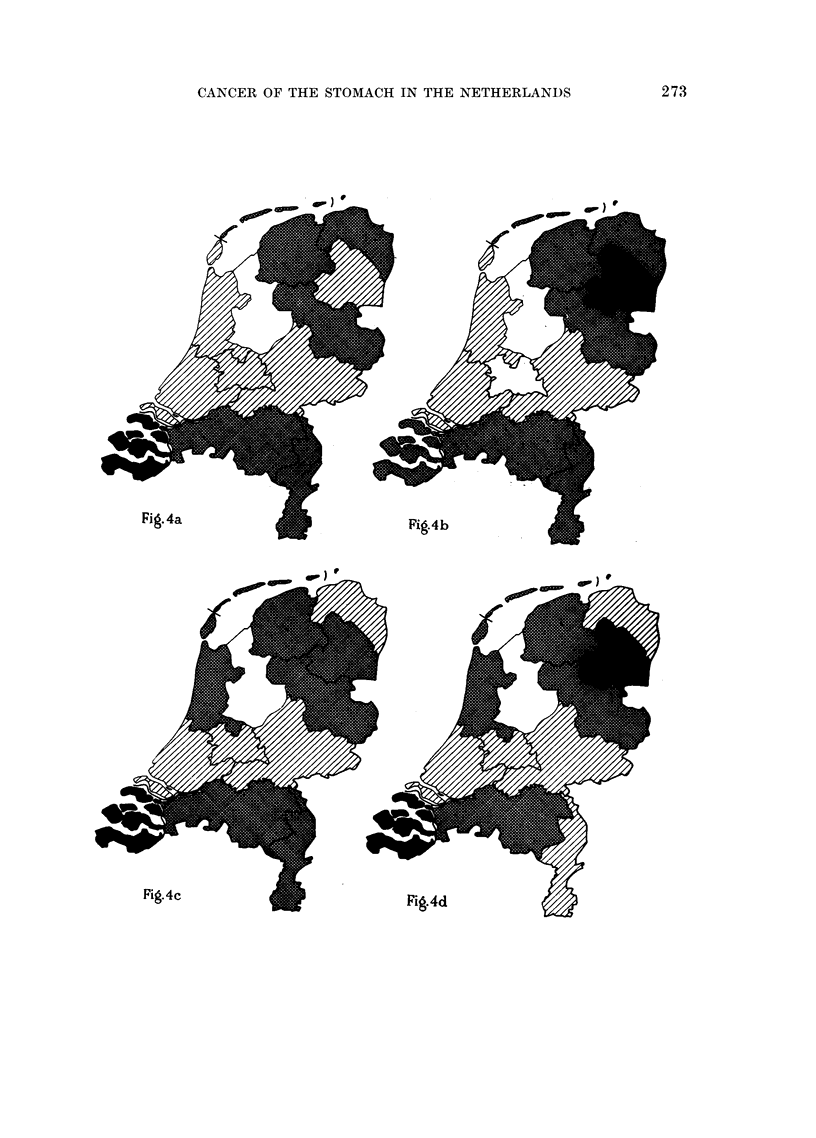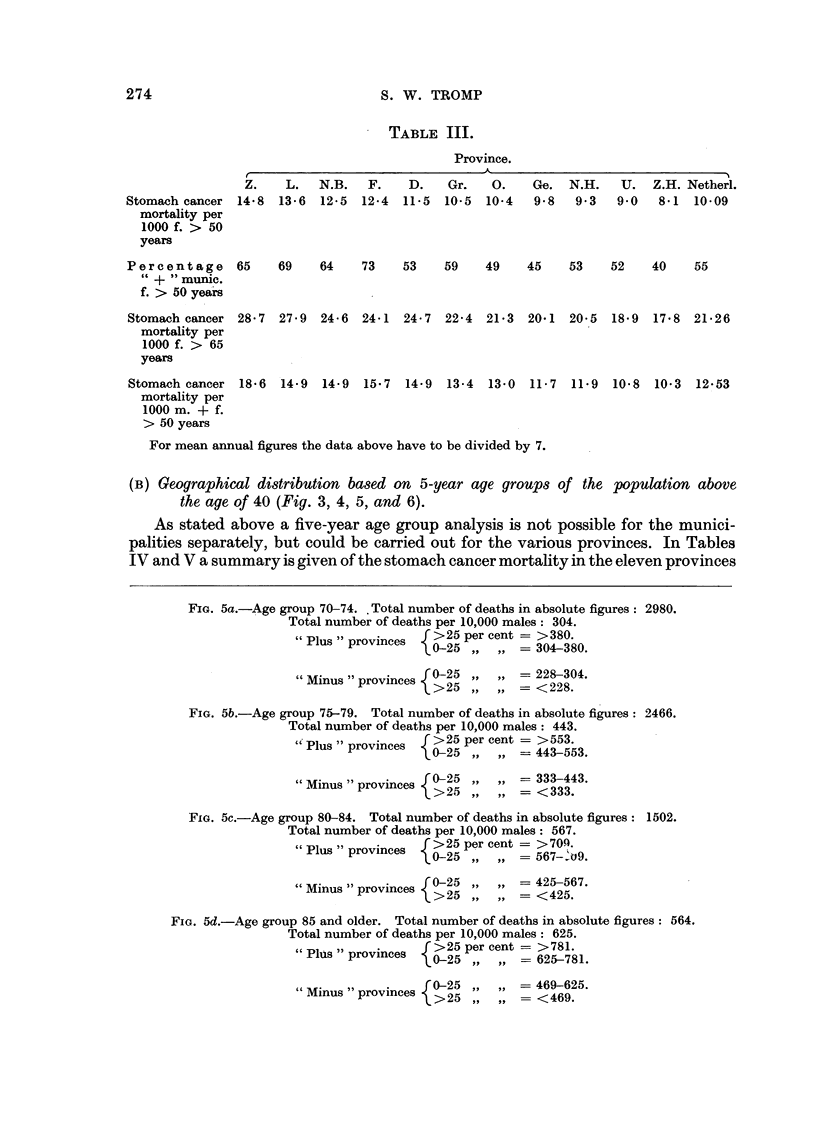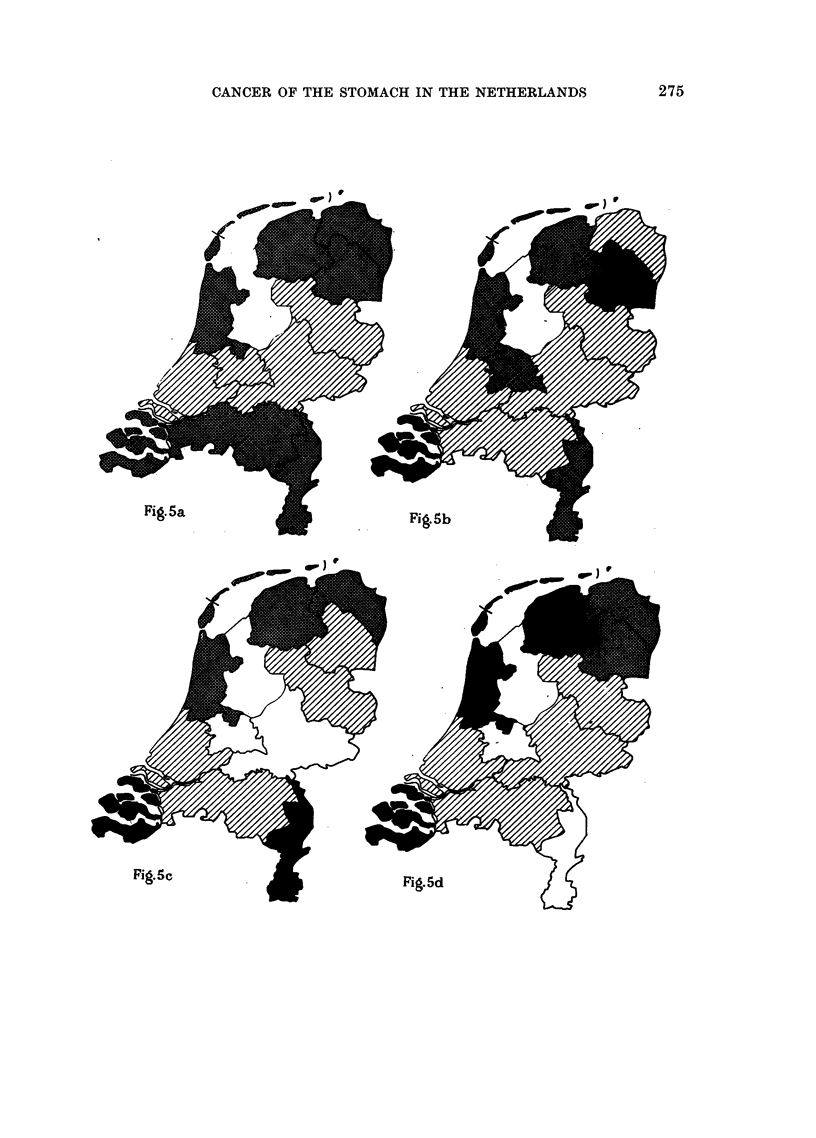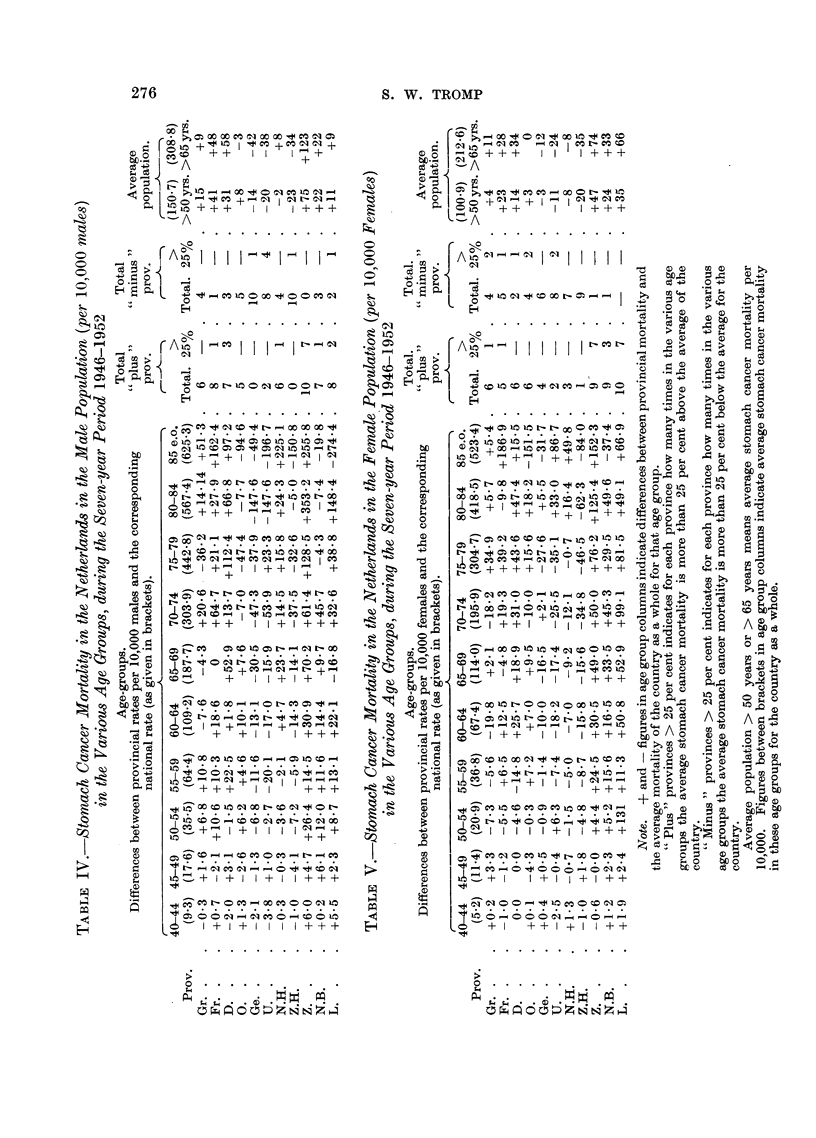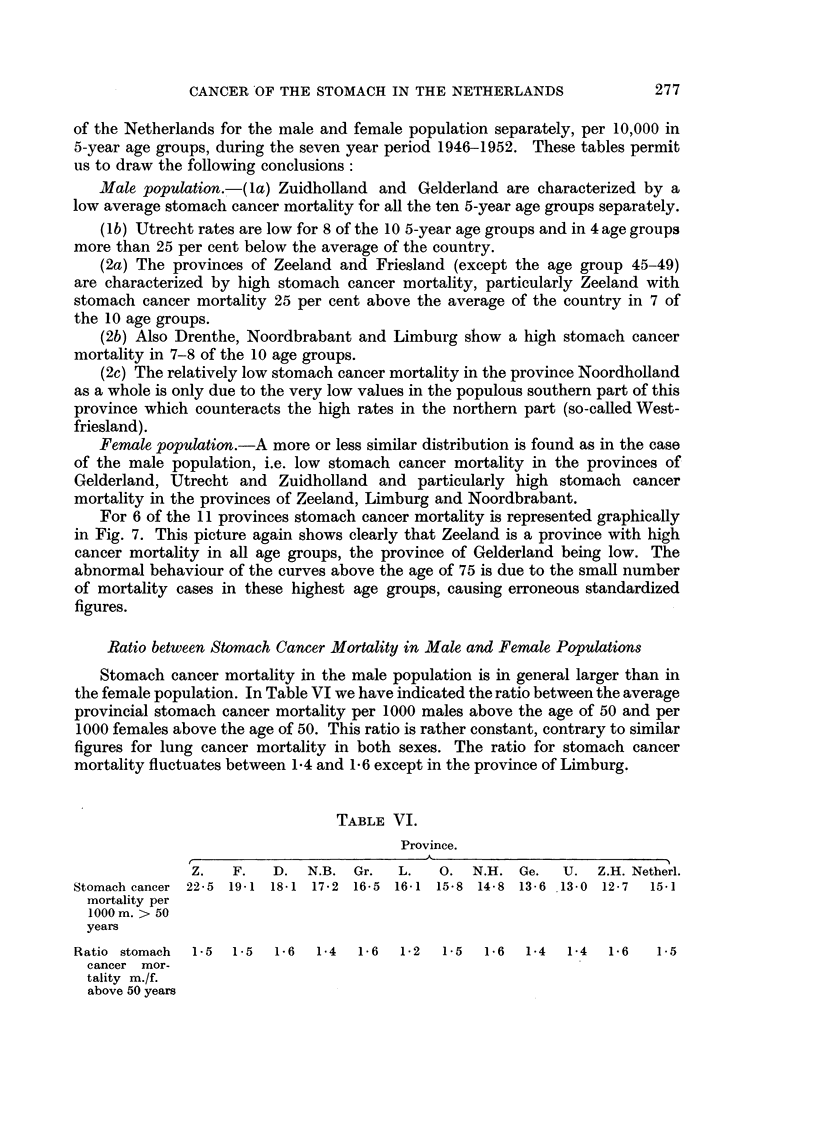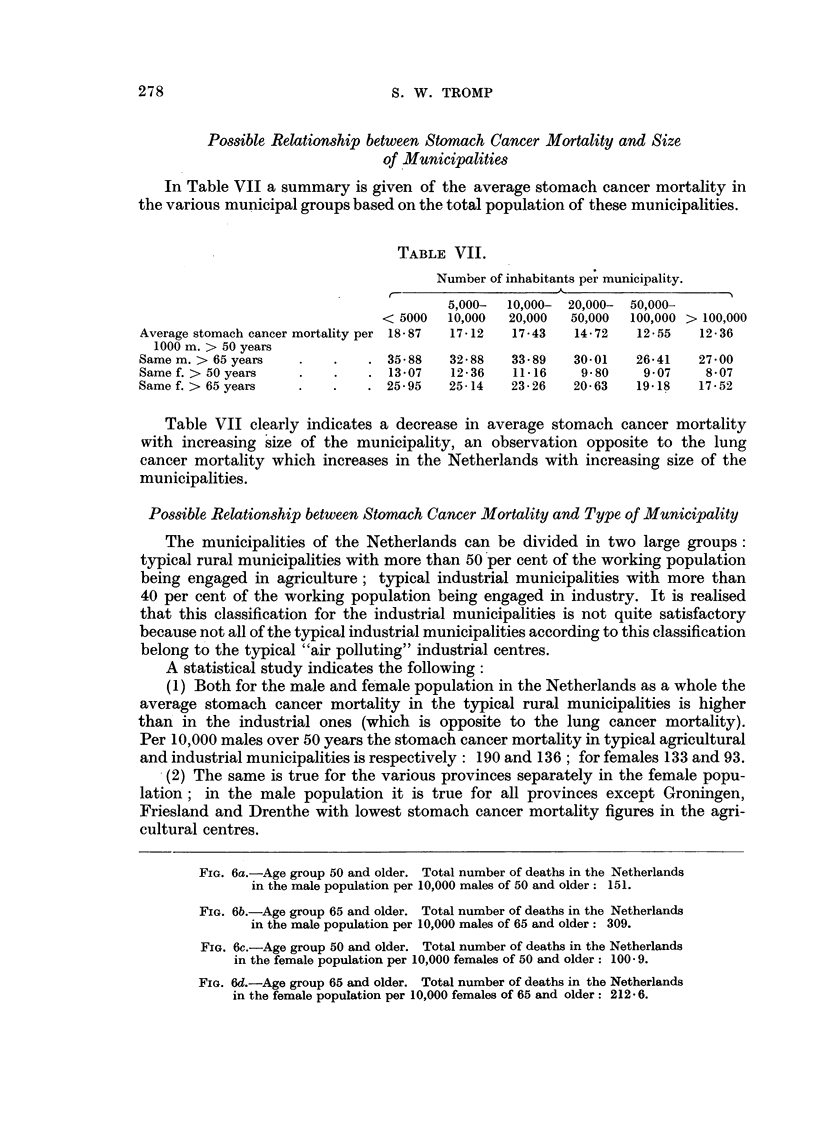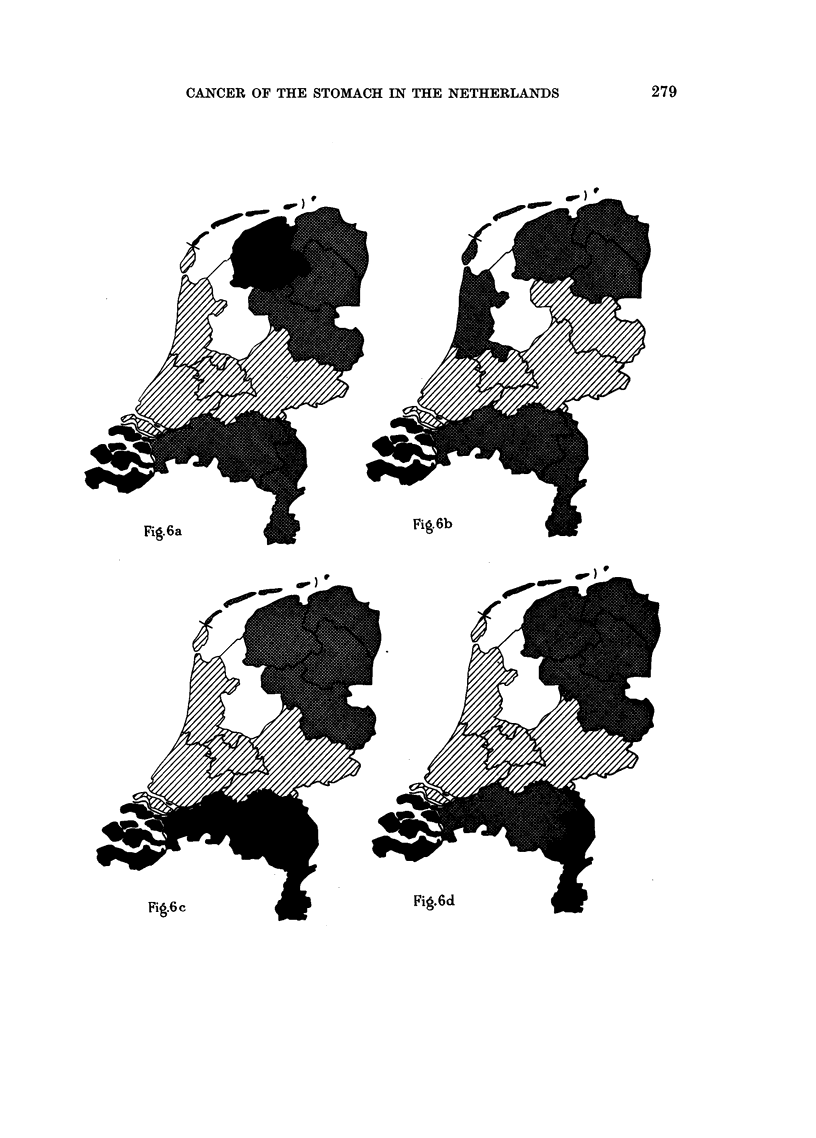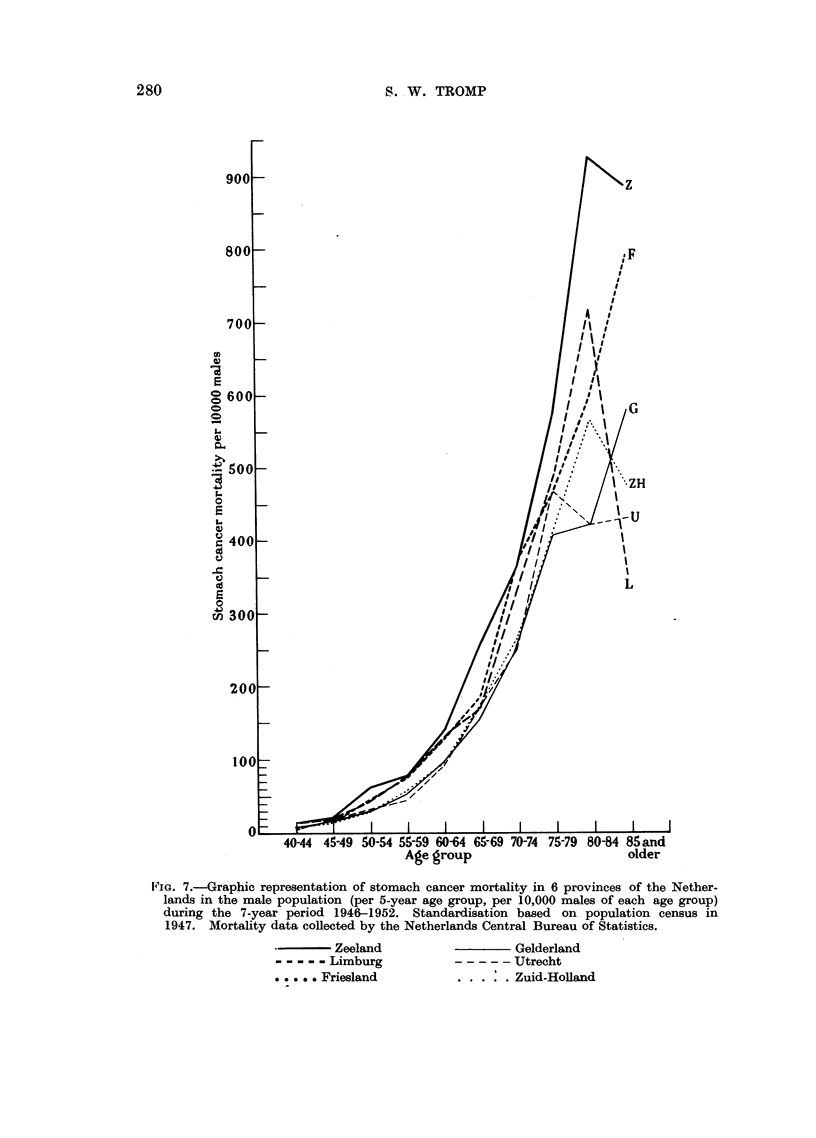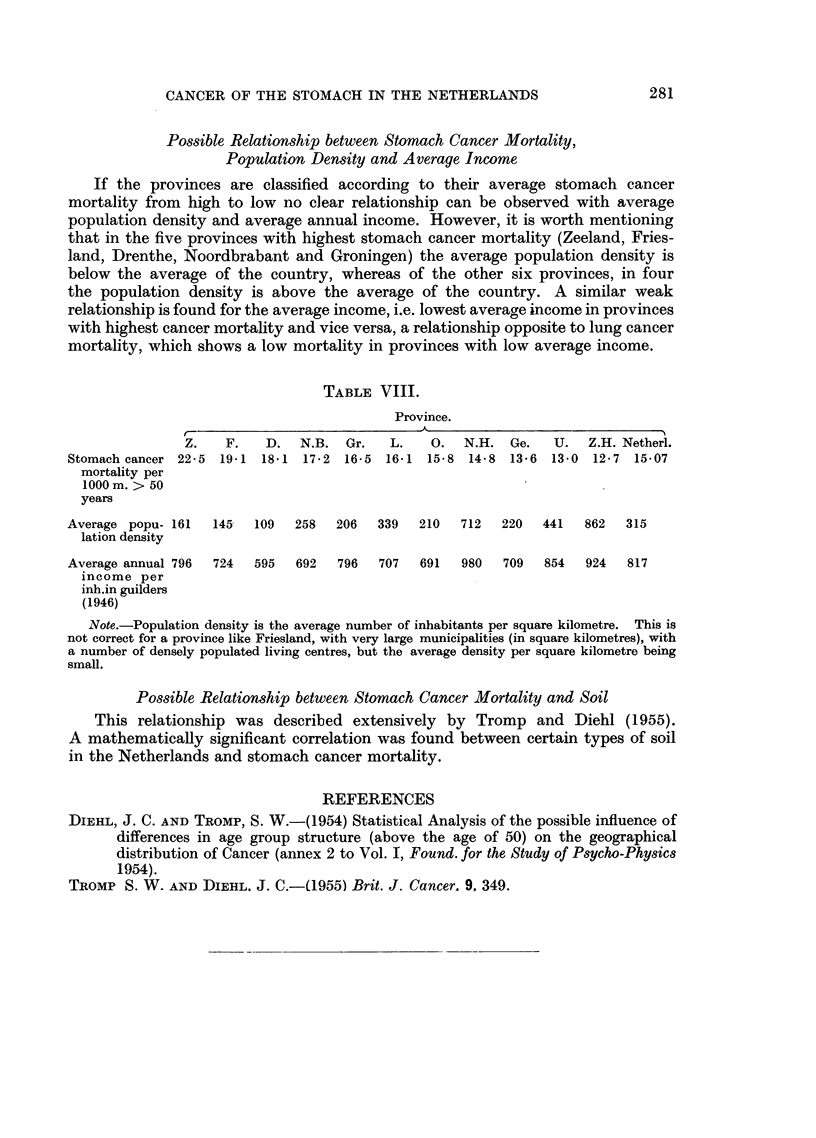# The Geographical Distribution of Cancer of the Stomach in the Netherlands (Period 1946-1952)

**DOI:** 10.1038/bjc.1956.31

**Published:** 1956-06

**Authors:** S. W. Tromp


### THE GEOGRAPHICAL DISTRIBUTION OF CANCER OF THE STOMACH IN THE NETHERLANDS (PERIOD 1946–1952)

S. W. TROMP

*From Hofbrouckerlaan* 54, *Oegstgeest* (*Leiden*), *Holland*

Received for publication February 23, 1956

THE various problems in relation to the study of the causes of cancer of the stomach could be approached in two principally different ways :

(1) *Laboratory, genetic and clinical research,* in particular cytological, histological, biochemical and other related studies.

(2) *Medical-geographical research :* starting from the geographical distribution of cancer of the stomach in the Netherlands, the deeper causes of this distribution could be studied which possibly could give a clue to the cause of cancer of the stomach as such.

### *Sources of Information*

As no morbidity data are available from all parts of the Netherlands the present study is based only on mortality data collected by the Netherlands Central Bureau of Statistics. In the present study only the period 1946–1952 was taken into consideration giving the best picture of the present stomach cancer mortality in the Netherlands. This period also has the advantage that in 1947 a population census took place which enabled us to calculate the mortality figures very accurately. For the seven years period 1946–1952 rates were obtained for the various 5-year age groups and classified according to sex, year and municipality.

The Netherlands Central Bureau of Statistics includes in the stomach cancer mortality figures : cancer of the stomach, cardia, pylori, papilla vateri, duodenum, sarcoma of the stomach, scirrhus. In view of the very small figures for most of these separate localisations and considering the uncertainty of diagnosis in many of these cases it was considered of greater value to combine these figures into one stomach-cancer-mortality figure.

### *Medical-geographical Methods Used*

The mortality figures, classified according to sex and age groups for the various municipalities (smallest administrative units in the Netherlands), were presented in various municipal maps by using a special colouring system indicating various groups of stomach cancer mortality frequency (Fig. 1 and 2). It is possible that small differences between two neighbouring municipalities (particularly small ones) are only caused by differences in diagnosis or age group structure above 50 years of age. However, if a large group of municipalities, either bordering each other or being close together, shows considerably higher cancer frequency figures than a similar large group of municipalities in another part of the province it is not very likely that only differences in diagnosis or age group structure can be considered to be the cause of the observed difference.

As indicated in Fig. 1 and 2 the geographical distribution of cancer mortality was not indicated for each 5-year age group separately, but the total mortality in the age groups 50 years and older was taken into consideration, because the municipal figures for the 5-year age groups are usually too small to be of any statistical significance. Five-year age group maps could be made only for the provincial distribution as indicated in Fig. 3, 4, 5 and 6. As differences in age group structure above the age of 50 could influence considerably the observed geographical distribution, apart from the maps based on the population above 50 years of age, similar maps were prepared on the basis of the population of 65 and older. For the age group 75 and older it was considered of no value to prepare such maps, as the observed differences have no statistical significance any more. The author realises that despite this age group correction still certain mistakes could occur in our maps. However, for our regional studies those possible errors have little importance for the following reasons.:

(1) As indicated in Fig. 1 and 2 the frequency groups lie rather far apart, ranging from 0–10 to 40 and over per 1000.

(2) Observed differences are only considered to be of any importance if the cancer frequencies in a group of neighbouring municipalities are more than 50 per cent above the average of the country.

Groups of municipalities with stomach cancer mortalities 50 per cent and more above the average of the country are called " PLUS AREAS ", those 25 per cent and more below the average of the country are indicated as " MINUS AREAS ".

Altogether six geographical distribution maps were prepared: two for the male population above 50 and above 65 years of age, two for the female population and two for the total population above 50 and 65 years of age. Only two of these maps are reproduced in this report (Fig. 1 and 2).

---

FIG. 1.—Total number of males, above the age of 50, who died from stomach cancer, per municipality, per 1000 male inhabitants above the age of 50. Average stomach cancer mortality in the Netherlands : 15·07 per 1000 males above the age of 50 years. (Based on data of the Central Bureau of Statistics in the Netherlands for the period 1946–1952.)

*Notes :* 1. " Stomach cancer mortality " comprises all deaths recorded by the Central Bureau of Statistics under the headings : cancer of the stomach, cardia, pylorus papilla vateri, duodenum, sarcoma of the stomach, scirrhus. 2. The municipalities Elten (Gelderland) and Tuddern (Limburg) were not included in our data because no data are available yet. 3. For all population data (number of inhabitants > 50 and > 65 years) the population census 1947 was used.

Scale = 1 : 400,000.

| | | | |
|---|---|---|---|
| Group 1 = > 40 | stomach cancer deaths (during 7 years) per 1000 males above the age of 50. | " Plus " areas. |
| " 2 = 31–40 | Ditto | |
| " 3 = 21–30 | " | |
| " 4 = 16–20 | " | |
| " 5 = 11–15 | " | " Minus " areas. |
| " 6 = 0–10 | " | |

Fig.1

### *Possible Effect of Differences in Age Groups Structure Above 50 Years of Age*

Despite our age group corrections (above 50 and above 65 years of age) still a number of physicians are inclined to believe that the greatest part of the observed geographical differences are only due to local differences in age group structure above 50 or 65. In a former publication (Diehl and Tromp, 1954) the various analyses made by the authors were summarized which contradict this assumption. If a large number of municipalities are classified according to their average stomach cancer frequency (on the basis of the population over 50) from high to low and the above mentioned assumption were true, we would expect a decrease of the percentages of population over 65 in these municipalities. Our studies clearly indicated that such a relation does not exist; in other words, a small part of the observed differences may be due to differences in age group structure above 65, but the latter cannot explain the main part of the observed differences.

Further support for this conclusion is given by the fact that the regional distribution observed in the maps based on the population over 50 is practically the same as the one in the maps based on the population over 65.

A second argument in favour of the statement above is the observation that in case of the provincial distribution maps (Fig. 3, 4, 5 and 6), which were made both for separate 5 year age groups after age 40 and for the total population of over 50 and 65 respectively, the same general distribution was observed. Further details of this study will be discussed later on in this report.

### *The Magnitude of the Mortality Figures*

The data used in the various maps are based on 14,044 deaths (during the period of seven years) in the male population above 50 years of age and 10,021 deaths in the female population of the same age group. For the population above 65 these figures are respectively 10,063 and 7,597. The provincial distribution of these figures is as follows:

TABLE I.

| | Province. | | | | | | | | | | |
|---|---|---|---|---|---|---|---|---|---|---|---|
| | Gr. | F. | D. | O. | Ge. | U. | N.H. | Z.H. | Z. | N.B. | L. |
| Males > 50 years | 773 | 965 | 457 | 977 | 1372 | 681 | 2711 | 2835 | 685 | 1681 | 907 |
| Females > 50 years | 526 | 642 | 278 | 634 | 1010 | 546 | 1907 | 2032 | 464 | 1212 | 770 |

FIG. 2.—Total number of females, above the age of 50, who died from stomach cancer, per municipality, per 1000 female inhabitants above the age of 50. Average stomach cancer mortality in the Netherlands: 10·09 per 1000 females above the age of 50 years. (Based on data of the Central Bureau of Statistics in the Netherlands for the period 1946–1952.)

See Notes 1, 2 and 3 for Fig. 1.

Scale = 1 : 400,000.

| | | | |
|---|---|---|---|
| — | Group 1 = > 40 | stomach cancer deaths (during 7 years) per 1000 females above the age of 50. | "Plus" areas. |
| — | „ 2 = 31–40 | Ditto | |
| — | „ 3 = 21–30 | „ | |
| — | „ 4 = 16–20 | „ | |
| — | „ 5 = 11–15 | „ | |
| — | „ 6 = 0–10 | „ | "Minus" areas' |

Fig. 2

FIG. 3, 4, 5, 6.—Geographical distribution of stomach cancer mortality in the 11 provinces of the Netherlands in the male population (per 5-year age group, per 10,000 males of each age group) during the 7-year period 1946–1952. (Standardisation based on population census in 1947.) (Mortality data collected by the Central Bureau of Statistics of the Netherlands.)

| | | |
|---|---|---|
| (black) — | Cancer mortality >25 per cent | Above the average for the country. |
| (dotted) — | „ „ 0–25 „ „ | |
| (hatched) — | „ „ 0–25 „ „ | Below the average for the country. |
| (white) — | „ „ >25 „ „ | |

FIG. 3*a*.—Age group 40–44. Total number of deaths in absolute figures : 278.
Total number of deaths per 10,000 males : 9·3.

" Plus " provinces {>25 per cent =11·6. ; 0–25 „ „ =9·3–11·6.

" Minus " provinces {0–25 „ „ =7·0–9·3. ; >25 „ „ =<7·0.

FIG. 3*b*.—Age group 45–49. Total number of deaths in absolute figures : 457.
Total number of deaths per 10,000 males : 18.

" Plus " provinces {>25 per cent = >22·5. ; 0–25 „ „ = 18–22·5.

" Minus " provinces {0–25 „ „ = 13·5–18. ; >25 „ „ = <13·5.

### *Problem of Increase and Decrease of Stomach Cancer Mortality*

During the period 1903--1930 the average yearly stomach cancer mortality in the Netherlands per 10,000 of the population (males and females together) over 40 was rather constant. It fluctuated between 22 and 24, with highest values in 1911 and 1913 (24·1). These mortality figures are based on the total cancer mortality of the stomach and liver together because in that period these two localisations were not separated. These figures are particularly untrustworthy if the cancer mortality is considered for each sex separately because liver cancer mortality in the female population is almost twice as high as in the case of the male population.

After 1930 when cancer mortality of the stomach and of the duodenum (without liver) was distinguished the average yearly rate was reduced to 15 per 10,000 of the population. During the period 1931–1949 the following decrease in stomach cancer mortality can be observed :

15·40 (in 1931)–15·76–15·10–14·58–14·65–14·81–14·63–14·67–14·01–14·36 (1940) –13·66–13·23–13·16–12·85–12·12 (1945)–10·82–11·32 11·31–11·98 (1949).

The observed decrease could be a true one, but may be partly due to various causes not related to cancer as such. For example it is possible that in the first years after 1930 a number of physicians did not separate sufficiently cancer of the liver and of the stomach in their death certificates. Future studies may reveal whether the observed decrease is a true one.

### *General Geographical Distribution of Cancer of the Stomach in the Netherlands*

(A) *Geographical distribution based on the population over* 50 *and* 65 *years of Age:*

*Male population.*—Stomach cancer mortality of the male population is about 1·5 times as high as that of the female population. However, apart from this difference the following summary will indicate a number of similarities in the geographical distribution for the two sexes.

High stomach cancer mortality is observed in the provinces of Groningen, Friesland S.W. part of Drenthe, a N.S. strip in the middle part of Overijssel, the northern part of Noordholland (so-called Westfriesland), the eastern part of Noordbrabant, the northern part of Limburg and particularly high values in the province of Zeeland (300–600 per cent above the average of the country).

Low stomach cancer mortality is found particularly in the southern part of the province of Gelderland (so-called Betuwe), in the area north of the Rhine-Maas river district (in the provinces of Gelderland, Utrecht and Zuidholland) and in the southern part of the province of Noordholland.

In Table II the average stomach cancer mortality for the various provinces based on the male population over 50 and 65 years of age is indicated. For each of these provinces the percentages of municipalities were determined with average cancer mortality above the average of the country (so-called " + " municipalities) and below this average (so-called " — " municipalities).

*Female population.*—High stomach cancer mortality is found in the provinces of Groningen, Friesland S.W. part of Drenthe, N.S. strip in the middle part of Overijssel, the northern part of Noordholland, the eastern part of Noordbrabant, the province of Limburg and particularly high values in the province of Zeeland.

TABLE II.

| | Province. | | | | | | | | | | | |
|---|---|---|---|---|---|---|---|---|---|---|---|---|
| | Z. | F. | D. | N.B. | Gr. | L. | O. | N.H. | Ge. | U. | Z.H. | Netherl. |
| Stomach cancer mortality per 1000 m. > 50 years | 22·5 | 19·1 | 18·1 | 17·2 | 16·5 | 16·1 | 15·8 | 14·8 | 13·6 | 13·0 | 12·7 | 15·07 |
| Percentage " + " munic. m. > 50 years | 70 | 75 | 62 | 67 | 70 | 58 | 53 | 67 | 43 | 57 | 46 | 59 |
| Stomach cancer mortality per 1000 m. > 65 years | 43·2 | 35·7 | 36·6 | 33·0 | 31·8 | 31·8 | 30·6 | 32·6 | 26·6 | 27·1 | 27·5 | 30·88 |
| Percentage " + " munic. m. > 65 years | 65 | 64 | 59 | 51 | 59 | 39 | 38 | 68 | 34 | 55 | 46 | 51 |

*Note :* The figures above represent the average stomach cancer mortality during seven years. For mean annual figures the figures above must be divided by 7.

In other words, the same distribution is found as in the case of the male population, only in the province of Limburg the difference between the mortality frequency of males and females, is smaller than in the other provinces.

Low stomach cancer mortality frequencies are found in the same areas as in the case of the male population.

---

FIG. 4*a*.—Age group 50–54. Total number of deaths in absolute figures : 842.
Total number of deaths per 10,000 males : 35·5.
" Plus " provinces { >25 per cent = >53. ; 0–25 ,, ,, = 35–53. }
" Minus " provinces { 0–25 ,, ,, = 18–35. ; >25 ,, ,, = <18. }

FIG. 4*b*.—Age group 55–59. Total number of deaths in absolute figures : 1293.
Total number of deaths per 10,000 males : 64.
" Plus " provinces { >25 per cent = >80. ; 0–25 ,, ,, = 64–80. }
" Minus " provinces { 0–25 ,, ,, = 48–64. ; >25 ,, ,, = <48. }

FIG. 4*c*.—Age group 60–64. Total number of deaths in absolute figures : 1857.
Total number of deaths per 10,000 males : 109.
" Plus " provinces { >25 per cent = >136. ; 0–25 ,, ,, = 109–136. }
" Minus " provinces { 0–25 ,, ,, = 82–109. ; >25 ,, ,, = <82. }

FIG. 4*d*.—Age group 65–69. Total number of deaths in absolute figures : 2568.
Total number of deaths per 10,000 males : 188.
" Plus " provinces { >25 per cent = >235. ; 0–25 ,, ,, = 188–235. }
" Minus " provinces { 0–25 ,, ,, = 141–188. ; >25 ,, ,, = <141. }

Fig. 4a

Fig. 4b

Fig. 4c

Fig. 4d

TABLE III.

| | Province. | | | | | | | | | | | |
|---|---|---|---|---|---|---|---|---|---|---|---|---|
| | Z. | L. | N.B. | F. | D. | Gr. | O. | Ge. | N.H. | U. | Z.H. | Netherl. |
| Stomach cancer mortality per 1000 f. > 50 years | 14·8 | 13·6 | 12·5 | 12·4 | 11·5 | 10·5 | 10·4 | 9·8 | 9·3 | 9·0 | 8·1 | 10·09 |
| Percentage " + " munic. f. > 50 years | 65 | 69 | 64 | 73 | 53 | 59 | 49 | 45 | 53 | 52 | 40 | 55 |
| Stomach cancer mortality per 1000 f. > 65 years | 28·7 | 27·9 | 24·6 | 24·1 | 24·7 | 22·4 | 21·3 | 20·1 | 20·5 | 18·9 | 17·8 | 21·26 |
| Stomach cancer mortality per 1000 m. + f. > 50 years | 18·6 | 14·9 | 14·9 | 15·7 | 14·9 | 13·4 | 13·0 | 11·7 | 11·9 | 10·8 | 10·3 | 12·53 |

For mean annual figures the data above have to be divided by 7.

(B) *Geographical distribution based on 5-year age groups of the population above the age of* 40 (*Fig.* 3, 4, 5, *and* 6).

As stated above a five-year age group analysis is not possible for the municipalities separately, but could be carried out for the various provinces. In Tables IV and V a summary is given of the stomach cancer mortality in the eleven provinces

---

FIG. 5*a*.—Age group 70–74. Total number of deaths in absolute figures : 2980.
Total number of deaths per 10,000 males : 304.
" Plus " provinces { >25 per cent = >380.
0–25 " " = 304–380.
" Minus " provinces { 0–25 " " = 228–304.
>25 " " = <228.

FIG. 5*b*.—Age group 75–79. Total number of deaths in absolute figures : 2466.
Total number of deaths per 10,000 males : 443.
" Plus " provinces { >25 per cent = >553.
0–25 " " = 443–553.
" Minus " provinces { 0–25 " " = 333–443.
>25 " " = <333.

FIG. 5*c*.—Age group 80–84. Total number of deaths in absolute figures : 1502.

Total number of deaths per 10,000 males : 567.

" Plus " provinces { >25 per cent = >709.

0–25 " " = 567–709.

" Minus " provinces { 0–25 " " = 425–567.

>25 " " = <425.

FIG. 5*d*.—Age group 85 and older. Total number of deaths in absolute figures : 564.
Total number of deaths per 10,000 males : 625.
" Plus " provinces { >25 per cent = >781.
0–25 " " = 625–781.
" Minus " provinces { 0–25 " " = 469–625.
>25 " " = <469.

of the Netherlands for the male and female population separately, per 10,000 in 5-year age groups, during the seven year period 1946–1952. These tables permit us to draw the following conclusions :

*Male population.*—(1*a*) Zuidholland and Gelderland are characterized by a low average stomach cancer mortality for all the ten 5-year age groups separately.

(1*b*) Utrecht rates are low for 8 of the 10 5-year age groups and in 4 age groups more than 25 per cent below the average of the country.

(2*a*) The provinces of Zeeland and Friesland (except the age group 45–49) are characterized by high stomach cancer mortality, particularly Zeeland with stomach cancer mortality 25 per cent above the average of the country in 7 of the 10 age groups.

(2*b*) Also Drenthe, Noordbrabant and Limburg show a high stomach cancer mortality in 7–8 of the 10 age groups.

(2*c*) The relatively low stomach cancer mortality in the province Noordholland as a whole is only due to the very low values in the populous southern part of this province which counteracts the high rates in the northern part (so-called West-friesland).

*Female population.*—A more or less similar distribution is found as in the case of the male population, i.e. low stomach cancer mortality in the provinces of Gelderland, Utrecht and Zuidholland and particularly high stomach cancer mortality in the provinces of Zeeland, Limburg and Noordbrabant.

For 6 of the 11 provinces stomach cancer mortality is represented graphically in Fig. 7. This picture again shows clearly that Zeeland is a province with high cancer mortality in all age groups, the province of Gelderland being low. The abnormal behaviour of the curves above the age of 75 is due to the small number of mortality cases in these highest age groups, causing erroneous standardized figures.

### *Ratio between Stomach Cancer Mortality in Male and Female Populations*

Stomach cancer mortality in the male population is in general larger than in the female population. In Table VI we have indicated the ratio between the average provincial stomach cancer mortality per 1000 males above the age of 50 and per 1000 females above the age of 50. This ratio is rather constant, contrary to similar figures for lung cancer mortality in both sexes. The ratio for stomach cancer mortality fluctuates between 1·4 and 1·6 except in the province of Limburg.

TABLE VI.

| | Province. | | | | | | | | | | | |
|---|---|---|---|---|---|---|---|---|---|---|---|---|
| | Z. | F. | D. | N.B. | Gr. | L. | O. | N.H. | Ge. | U. | Z.H. | Netherl. |
| Stomach cancer mortality per 1000 m. > 50 years | 22·5 | 19·1 | 18·1 | 17·2 | 16·5 | 16·1 | 15·8 | 14·8 | 13·6 | 13·0 | 12·7 | 15·1 |
| Ratio stomach cancer mortality m./f. above 50 years | 1·5 | 1·5 | 1·6 | 1·4 | 1·6 | 1·2 | 1·5 | 1·6 | 1·4 | 1·4 | 1·6 | 1·5 |

*Possible Relationship between Stomach Cancer Mortality and Size of Municipalities*

In Table VII a summary is given of the average stomach cancer mortality in the various municipal groups based on the total population of these municipalities.

TABLE VII.

| | Number of inhabitants per municipality. | | | | | |
|---|---|---|---|---|---|---|
| | < 5000 | 5,000–10,000 | 10,000–20,000 | 20,000–50,000 | 50,000–100,000 | > 100,000 |
| Average stomach cancer mortality per 1000 m. > 50 years | 18·87 | 17·12 | 17·43 | 14·72 | 12·55 | 12·36 |
| Same m. > 65 years | 35·88 | 32·88 | 33·89 | 30·01 | 26·41 | 27·00 |
| Same f. > 50 years | 13·07 | 12·36 | 11·16 | 9·80 | 9·07 | 8·07 |
| Same f. > 65 years | 25·95 | 25·14 | 23·26 | 20·63 | 19·18 | 17·52 |

Table VII clearly indicates a decrease in average stomach cancer mortality with increasing size of the municipality, an observation opposite to the lung cancer mortality which increases in the Netherlands with increasing size of the municipalities.

*Possible Relationship between Stomach Cancer Mortality and Type of Municipality*

The municipalities of the Netherlands can be divided in two large groups: typical rural municipalities with more than 50 per cent of the working population being engaged in agriculture; typical industrial municipalities with more than 40 per cent of the working population being engaged in industry. It is realised that this classification for the industrial municipalities is not quite satisfactory because not all of the typical industrial municipalities according to this classification belong to the typical "air polluting" industrial centres.

A statistical study indicates the following:

(1) Both for the male and female population in the Netherlands as a whole the average stomach cancer mortality in the typical rural municipalities is higher than in the industrial ones (which is opposite to the lung cancer mortality). Per 10,000 males over 50 years the stomach cancer mortality in typical agricultural and industrial municipalities is respectively: 190 and 136; for females 133 and 93.

(2) The same is true for the various provinces separately in the female population; in the male population it is true for all provinces except Groningen, Friesland and Drenthe with lowest stomach cancer mortality figures in the agricultural centres.

---

FIG. 6*a*.—Age group 50 and older. Total number of deaths in the Netherlands in the male population per 10,000 males of 50 and older: 151.

FIG. 6*b*.—Age group 65 and older. Total number of deaths in the Netherlands in the male population per 10,000 males of 65 and older: 309.

FIG. 6*c*.—Age group 50 and older. Total number of deaths in the Netherlands in the female population per 10,000 females of 50 and older: 100·9.

FIG. 6*d*.—Age group 65 and older. Total number of deaths in the Netherlands in the female population per 10,000 females of 65 and older: 212·6.

Fig. 6a

Fig. 6b

Fig. 6c

Fig. 6d

Fig. 7.—Graphic representation of stomach cancer mortality in 6 provinces of the Netherlands in the male population (per 5-year age group, per 10,000 males of each age group) during the 7-year period 1946–1952. Standardisation based on population census in 1947. Mortality data collected by the Netherlands Central Bureau of Statistics.

| | |
|---|---|
| ——— Zeeland | ——— Gelderland |
| - - - - Limburg | – – – – Utrecht |
| • • • • Friesland | . . . . . Zuid-Holland |

### *Possible Relationship between Stomach Cancer Mortality, Population Density and Average Income*

If the provinces are classified according to their average stomach cancer mortality from high to low no clear relationship can be observed with average population density and average annual income. However, it is worth mentioning that in the five provinces with highest stomach cancer mortality (Zeeland, Friesland, Drenthe, Noordbrabant and Groningen) the average population density is below the average of the country, whereas of the other six provinces, in four the population density is above the average of the country. A similar weak relationship is found for the average income, i.e. lowest average income in provinces with highest cancer mortality and vice versa, a relationship opposite to lung cancer mortality, which shows a low mortality in provinces with low average income.

TABLE VIII.

| | Province. | | | | | | | | | | | |
|---|---|---|---|---|---|---|---|---|---|---|---|---|
| | Z. | F. | D. | N.B. | Gr. | L. | O. | N.H. | Ge. | U. | Z.H. | Netherl. |
| Stomach cancer mortality per 1000 m. > 50 years | 22·5 | 19·1 | 18·1 | 17·2 | 16·5 | 16·1 | 15·8 | 14·8 | 13·6 | 13·0 | 12·7 | 15·07 |
| Average population density | 161 | 145 | 109 | 258 | 206 | 339 | 210 | 712 | 220 | 441 | 862 | 315 |
| Average annual income per inh.in guilders (1946) | 796 | 724 | 595 | 692 | 796 | 707 | 691 | 980 | 709 | 854 | 924 | 817 |

*Note.*—Population density is the average number of inhabitants per square kilometre. This is not correct for a province like Friesland, with very large municipalities (in square kilometres), with a number of densely populated living centres, but the average density per square kilometre being small.

### *Possible Relationship between Stomach Cancer Mortality and Soil*

This relationship was described extensively by Tromp and Diehl (1955). A mathematically significant correlation was found between certain types of soil in the Netherlands and stomach cancer mortality.

### REFERENCES

DIEHL, J. C. AND TROMP, S. W.—(1954) Statistical Analysis of the possible influence of differences in age group structure (above the age of 50) on the geographical distribution of Cancer (annex 2 to Vol. I, *Found. for the Study of Psycho-Physics* 1954).

TROMP S. W. AND DIEHL. J. C.—(1955) *Brit. J. Cancer.* **9**. 349.